# Application of Tamarind Shell as a Green Additive in Natural Rubber

**DOI:** 10.3390/polym16040493

**Published:** 2024-02-09

**Authors:** Weenusarin Intiya, Kannika Hatthapanit, Puchong Thaptong, Pongdhorn Sae-oui

**Affiliations:** National Metal and Materials Technology Center (MTEC), National Science and Technology Development Agency (NSTDA), Khlong Nueng, Khlong Luang, Pathum Thani 12120, Thailand; weenusai@mtec.or.th (W.I.); kannikh@mtec.or.th (K.H.); puchongt@mtec.or.th (P.T.)

**Keywords:** rubber, tamarind shell, eco-friendly, additives, properties

## Abstract

The feasibility of using tamarind shell as an eco-friendly additive in natural rubber (NR) was studied. Tamarind shell powder (TSP) was prepared with different particle size ranges before being characterized by various techniques such as Fourier transform infrared spectroscopy (FTIR), thermogravimetric analysis (TGA), elemental analysis, etc. The results of the FTIR and elemental analysis confirmed that TSP was mainly composed of amino acids (proteins), celluloses, and tannins. The thermal analysis revealed that TSP contained approximately 9% moisture, and its main constituents were stable up to 200 °C, which is higher than the normal processing temperature of rubber products. The addition of TSP to NR led to reductions in scorch time and cure time due to the presence of moisture and proteins. This phenomenon was more obvious with the decrease in TSP’s particle size. Even though the small addition of TSP (≤10 phr) did not cause any change in hardness, it significantly impaired the mechanical properties of the rubber vulcanizates, particularly tensile strength, elongation at break, and abrasion resistance. Such deterioration depended greatly on the TSP particle size, i.e., the finest particles (S-TSP) showed the least deterioration of mechanical properties. In summary, TSP can be considered a low-cost, eco-friendly bio-additive for rubbers. Nevertheless, it must be used with great care to avoid undesirable impacts on mechanical properties.

## 1. Introduction

Tamarind (*Tamrindus Indica* L.), a brown pod-like tropical fruit, is widely produced in many parts of the world, particularly in Africa, India, America, and Southeast Asia. Thailand has recently become one of the largest sweet tamarind producers, with an annual yield of around 100,000 tons. Among sweet tamarind cultivars, Sri Thong is one of the most popular species because, apart from its renowned sweetness, it has a large, long pod that usually contains up to 10 seeds embedded in the brown, edible pulp. When fully ripe, the pulp becomes juicy and turns dark brown, while the shells are brittle and easily broken. According to its huge annual consumption, an enormous amount of tamarind shell is produced annually because about 11–30% of the tamarind pod’s weight is made up of the shell [1,2]. This tamarind shell is generally regarded as an agricultural waste. Its major constituents are carbohydrates (celluloses), calcium carbonate (CaCO_3_), proteins, tannins, moisture, etc. [1]. Due to its carbon-rich composition, tamarind shell has been utilized as a biomass material in many countries [3]. As the tamarind shell is made up of calcium carbonate (CaCO_3_) ca. 11% by weight [4], the ash obtained from the biomass power plants is generally rich in calcium (Ca). Other elements such as potassium (K), magnesium (Mg), sodium (Na), zinc (Zn), manganese (Mn), iron (Fe), and other trace elements are also found [5]. Attempts have therefore been made to use biomass ash, including tamarind shell ash, as a low-cost and effective catalyst for the transesterification process and biodiesel production [6,7,8]. The literature also reports an abundance of polyphenolic compounds, including tannins, in tamarind shell [9,10]. Therefore, Abdullahi et al. studied the potential use of tannins extracted from tamarind shell in rust transformation [11]. Tamarind shell was also employed as a raw material in the preparation of bio-activated carbon nanosheets, a key component of energy storage devices, via the chemical vapor deposition (CVD) process [12]. Similarly, nitrogen-doped porous carbon nanosheets were prepared from tamarind shell via the pyrolysis method and used as an electrode material for the effective capacitive deionization (CDI) process [13]. Many works also reported the preparation and application of activated carbon from tamarind shell [14,15]. Recently, tamarind shell has been used as a filler in epoxy composites [16]. The addition of tamarind shell powder (TSP) showed considerable increases in hardness and flexural strength with the sacrifice of thermal stability of the epoxy composites. Modification of TSP by depositing metal nanoparticles onto its surface via a hydrothermal method has been revealed [17,18]. Both copper and silver nanoparticles were deposited. The modified TSP showed antibacterial activity against both Gram-positive and Gram-negative bacteria and hence can be used as filler in the preparation of antibacterial polymer composites. In addition, Suguna, et al. used the tamarind fruit nut shell powder as an adsorbent for the removal of manganese from aqueous solutions [19].

Despite a growing interest in using tamarind shell in many fields, the application of this agricultural waste in rubber fields has not been fully investigated. To fill the gap, this work aimed to study the feasibility of utilizing tamarind shell in rubber compounding. The tamarind shell was initially ground into a fine powder, characterized, and finally added to natural rubber (NR) in various amounts. The effect of the powder’s particle size on the properties of the rubber was also evaluated. Due to its relatively large particle size compared to the commonly used reinforcing filler, the maximum content of tamarind shell powder was limited to 10 phr to avoid excessive deterioration of the mechanical properties of the vulcanizates. The implementation of tamarind shell in rubber compounding not only broadens the application of this waste but also reduces rubber products’ costs.

## 2. Experimental Section

### 2.1. Materials

Tamarind shell was collected from a local vendor in Petchaboon Province, Thailand. The shell was taken from sweet tamarind (Sri Thong cultivar). Natural rubber (NR; STR 5L) was supplied by Union Rubber Products Corp., Ltd. (Bangkok, Thailand). Its basic properties are as follows: Mooney viscosity (ML(1 + 4)@100 °C) = 75.5 MU, initial plasticity number (P_0_) = 42, and plasticity retention index (PRI) = 93. The suppliers of the compounding ingredients are listed in Table 1.

### 2.2. Preparation and Characterization of Tamarind Shell Powder (TSP)

The tamarind shell was initially dried in an oven at 105 °C for 2 h prior to being ground into powder by an automatic grinder (Hok Tai Machinery, Samutprakan, Thailand) for 3 min. After grinding, the tamarind shell powder (TSP) was sieved through a series of screens, i.e., 40-mesh (~400 μm), 200-mesh (~74 μm), and 325-mesh (~44 μm), yielding three TSP samples with different particle sizes. The TSP left over on the 40-mesh screen was discarded. The TSP samples left over on the 200-mesh and 325-mesh screens were herein called L-TSP and M-TSP, respectively. The TSP sample passing through a 325-mesh screen was called S-TSP.

Determination of the functional groups of TSP was carried out using a Fourier transform infrared spectrophotometer (FTIR: Tensor 27, Bruker, Ettlingen, Germany) at a resolution of 4 cm^−1^ and scanning range of 4000–600 cm^−1^. The TSP sample was thoroughly mixed with potassium bromide (KBr) at a concentration of 0.8%wt. and pressed in a mold to form a thin circular disc prior to being analyzed. The thermal behavior of TSP was investigated using a thermogravimetric analyzer (TGA: Mettler Toledo, Melbourne, Australia). The temperature was scanned from 30 to 800 °C at a heating rate of 20 °C/min under a nitrogen atmosphere. Elemental analysis was carried out to determine the amount of carbon (C), hydrogen (H), oxygen (O), and nitrogen (N) by a CHNOS analyzer (628 Series, Leco Corporation, St. Joseph, MI, USA) based on the combustion method. In the analysis, carbon was converted to carbon dioxide, hydrogen to water, nitrogen to nitrogen gas or oxides of nitrogen, prior to the detection of the gases by various techniques. The average particle size of the TSP samples was measured by a Mastersizer-S (Malvern Panalytical, Malvern, UK). Moisture content was measured by measuring the weights of the samples before and after drying in an oven at 105 °C for 3 h. The measurement of the intrinsic density of the TSP samples was performed by an ultrapycnometer (Ultrapyc 1200e, Quantachrome Instruments, Boynton Beach, FL, USA). The pH value was also measured by mixing 1 g of the sample in 10 mL of deionized water and a few drops of acetone before measuring the pH value using a pH meter (pH-700, Apera Instruments, Columbus, OH, USA). Since all samples (S-TSP, M-TSP, and L-TSP) were prepared from the same lot and the particle size does not greatly affect the functional groups, thermal behavior, intrinsic density, pH, and elemental compositions of the samples, only the S-TSP sample was used for these characterizations. 

### 2.3. Rubber Compound Preparation and Testing

Various amounts of the prepared TSP samples were added to NR in accordance with the formulas given in Table 2. Mixing was performed in an internal mixer (Polylab OS, Thermo Scientific, Karlsruhe, Germany) under the following conditions: an initial temperature of 60 °C, a rotor rotation speed of 40 rpm, and a fill factor of 0.8. The mixing was carried out for 10 min. After mixing, the rubber compounds were dumped, sheeted on a two-roll mill, and left at room temperature overnight (≥16 h) before being tested.

The cure characteristics of the rubber compounds were evaluated at 150 °C for 20 min by a moving die rheometer (MDR: TechPRO MD+, CG Engineering Co., Ltd., Pathum Thani, Thailand) as per ISO 6502-3 [20]. The scorch time (t_s1_), defined as the time taken for the torque to rise from a minimum value to the specified increase value (1 dN.m), and the cure time (t_c_95) were recorded. Additionally, the maximum torque (M_H_) and minimum torque (M_L_) were also monitored. In this study, the torque difference (M_H_ − M_L_) was used to indirectly indicate the degree of crosslinking of the rubbers. After the cure test, the pre-determined cure time was used to prepare the 2 mm thick rubber vulcanizates in a hydraulic hot-press at 150 °C. The mechanical properties of the vulcanized rubbers were then investigated. The hardness test was carried out on a 6 mm thick specimen using a Shore A durometer (HT3000, MonTech Rubber Testing Solutions, Buchen, Germany) based on ISO 48-4 [21]. Tensile properties, including tensile strength and elongation at break, were evaluated using a universal testing machine (NRI-TS500-20B (Extra), Narin Instrument Co., Ltd., Samutprakan, Thailand) following ISO 37 [22]. Five dumbbell-shaped specimens (die type 1) were prepared and tested at a crosshead speed of 500 mm/min with a load cell of 1 kN. Abrasion resistance was evaluated using a DIN abrasion tester (GT7012DA, Gotech Testing Machine Inc., Taichung, Taiwan) in accordance with ISO 4649 Method A [23]. The weight loss after abrasion at a distance of 40 m was initially measured and later converted to volume loss by dividing by the specimen’s density. The accelerated aging test was performed in accordance with ISO 188 Method A [24]. The standard specimens were aged in a cabinet oven at 70 °C for 72 h prior to measuring their hardness and tensile properties. The aging resistance of the rubber vulcanizates was represented in terms of the percentage change in properties after the aging test. The equilibrium swelling test was performed by soaking the weighed rubber specimens (10 × 10 × 2 mm) in toluene at room temperature for 5 days. The weights of the rubber specimens were then measured immediately after removing excessive toluene from the specimens’ surfaces with filter paper. The swelling ratio at equilibrium state was then calculated. The rule of three was employed to normalize the effect of the variation in filler loading in the samples, i.e., the swelling ratio must be compared at the same swellable rubber content. The morphology of the rubber vulcanizates was determined using a scanning electron microscope (SEM: JSM7800F, JEOL Asia Pte. Ltd., Singapore). The tensile-fracture surfaces were coated with a thin layer of gold to alleviate an electron bombardment effect during the examination. The thermal stability of the rubber vulcanizates was determined by a thermogravimetric analyzer (TGA: Mettler Toledo, Melbourne, Australia) under a nitrogen atmosphere at a heating rate of 20 °C/min. The functional groups of the filled vulcanizates were evaluated by using an attenuated total reflectance Fourier transform infrared spectrophotometer (ATR-FTIR: Tensor 27, Bruker, Ettlingen, Germany) at a resolution of 4 cm^−1^ and a scanning range of 4000–600 cm^−1^.

## 3. Results and Discussion

### 3.1. Basic Characterization of TSP

**Figure 1:**
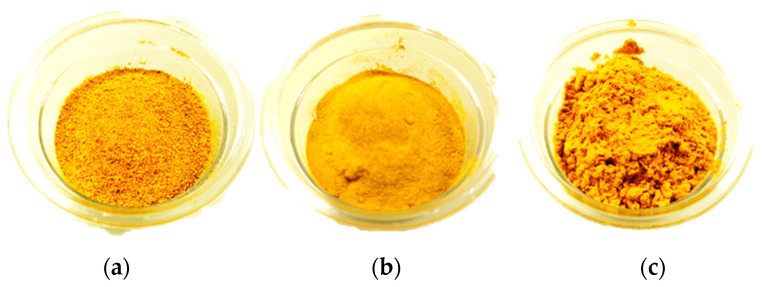
Appearance of (**a**) L-TSP, (**b**) M-TSP, and (**c**) S-TSP.

Figure 1 shows the appearance of the TSP samples (L-TSP, M-TSP, and S-TSP). Clearly, all samples are golden fine powder with different fineness levels.

The basic properties of the samples are given in Table 3. The average particle sizes of L-TSP, M-TSP, and S-TSP were 191.5, 29.8, and 17.2 μm, respectively. It is obvious that the average particle sizes of all samples are very high (on the microscale), even for the one passing through a 325-mesh screen (S-TSP). The moisture content of the samples varied in a small range from 5.7% to 6.7%, indicating the capability of TSP to adsorb moisture from the environment. As can be seen, L-TSP had the lowest moisture content, which could be explained by its largest particle size and the fact that it has the smallest surface area for moisture adsorption. According to the results in Table 3, the intrinsic density of TSP was ca. 1.56 g/cm^3^. The pH value of TSP was ca. 4.5, indicating that TSP was a weak acid due to the abundance of polyphenols [2,9,10,11,25]. The elemental analysis revealed that TSP consisted of approximately 48.0%, 45.6%, 5.7%, and 0.7% of carbon (C), oxygen (O), hydrogen (H), and nitrogen (N), respectively.

**Figure 2:**
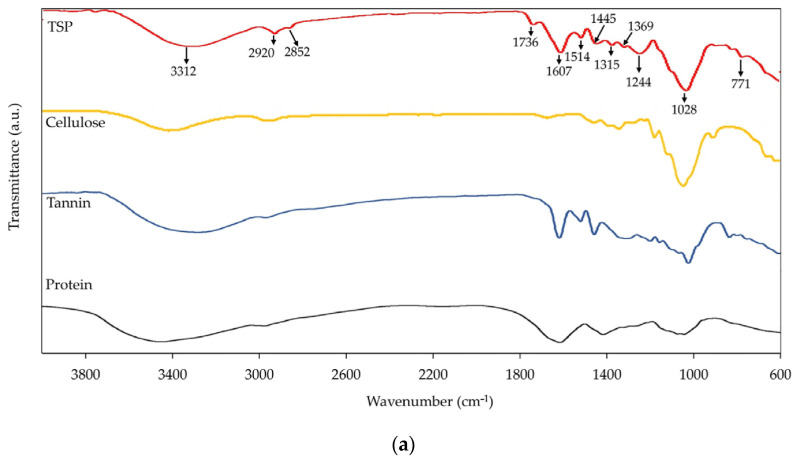

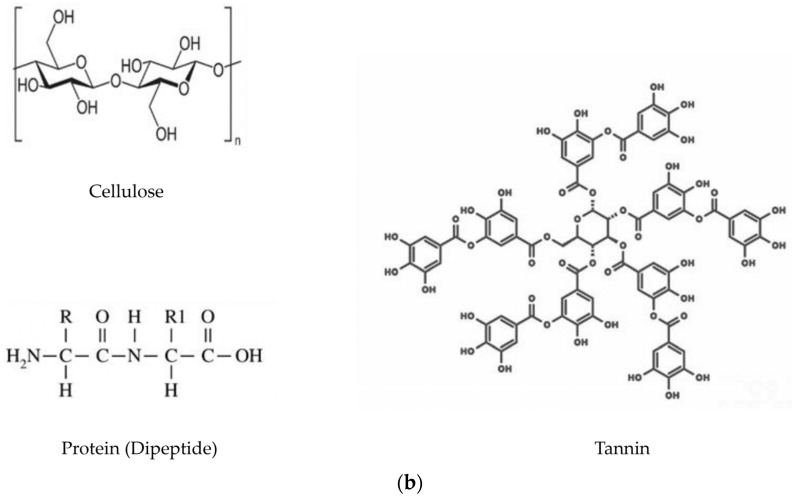
FTIR analysis: (**a**) FTIR spectra of TSP, cellulose, tannin, and protein and (**b**) chemical structures of the TSP’s main components.

Figure 2a shows the FTIR spectrum of the TSP sample. The absorption peak found at 3312 cm^−1^ is assigned to the stretching vibration of the O–H bond, mainly from polyphenols of tannins, hydroxyl groups of cellulose, and moisture adsorbed on the TSP [26]. The peaks at 2920 and 2852 cm^−1^ belong to the stretching vibrations of CH_2_ and CH bonds of aliphatic hydrocarbons, respectively [11]. The peak at 1736 cm^−1^ arises from the vibration of the C=O of carboxylic and ester groups. The peaks found at 1607, 1514, and 1445 cm^−1^ are the characteristic peaks of aromatic rings. In addition, the peak at 1607 cm^−1^ may occur as a result of the C=O vibration of amide group of proteins [27]. The peak at 1369 cm^−1^ is attributed to the O–H bending vibration of phenol. The absorption peaks observed at 1315, 1244, and 1028 cm^−1^ correspond to the symmetric stretching vibration of C–O–C. The FTIR results show that TSP contains a variety of functional groups such as hydroxyls, phenols, aromatic rings, amino acids, carboxylic acids, and C–O–C of celluloses. Such results are in good agreement with the literature, which reveals that the main compositions of tamarind shell are proteins, tannins, and celluloses [28]. Examples of FTIR spectra of cellulose, protein, and tannin are also included in Figure 2a, while the chemical structures of these components are given in Figure 2b.

**Figure 3:**
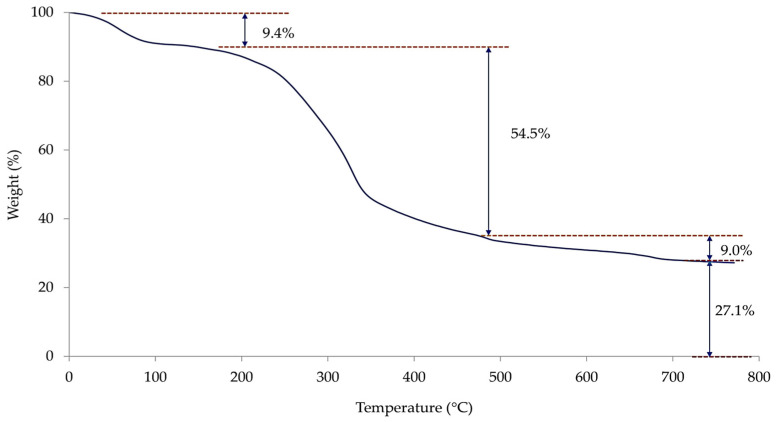
TGA thermogram of TSP.

The TGA thermogram of TSP is displayed in Figure 3. The thermal decomposition of TSP can be divided into various stages. The first stage was found at a temperature below 150 °C with approximately 9.4% of weight loss due to the evaporation of moisture adsorbed in the sample. Similar observations have been reported for other agricultural wastes [28,29,30]. Compared with the results in Table 3, the moisture content measured from TGA showed a slightly higher value, probably due to the continuous moisture adsorption taking place during the storage before the TGA testing. The second stage of weight loss, found at a temperature range of 150–480 °C, constituted around 54.5%, which is attributed to the decomposition of the main constituents of TSP, i.e., proteins, celluloses, and tannins. The overall weight loss at this stage of TSP was similar to that reported by Amit Amulani et al. [31]. The results revealed that the main constituents of TSP were stable up to almost 200 °C, which is generally higher than the processing temperature of rubber production.

The third stage was observed at a temperature range of 480–800 °C, where the weight slightly decreased with the increase in temperature due to the continuation of secondary decomposition reactions [31,32]. The overall weight loss at this stage was 9.0%. A significant amount of residue was also observed at the end of the pyrolysis (ca. 27.1%). As tannin, one of the main components of TSP, contains many aromatic rings in its chemical structure, it is difficult to achieve full decomposition during the pyrolysis under inert gas. In addition, nitrogen-containing compounds such as proteins in TSP can facilitate char formation during pyrolysis [33]. Similar observations have been reported for spent coffee grounds (SCGs) [34,35,36]. This residue is therefore a mixture of char and inorganic alkalis, particularly calcium carbonate (CaCO_3_), which is reported to be one of the main inorganic substances in tamarind shell [6,14,37].

### 3.2. Properties of Rubber Compounds and Vulcanizates

#### 3.2.1. Cure Characteristics

**Figure 4:**
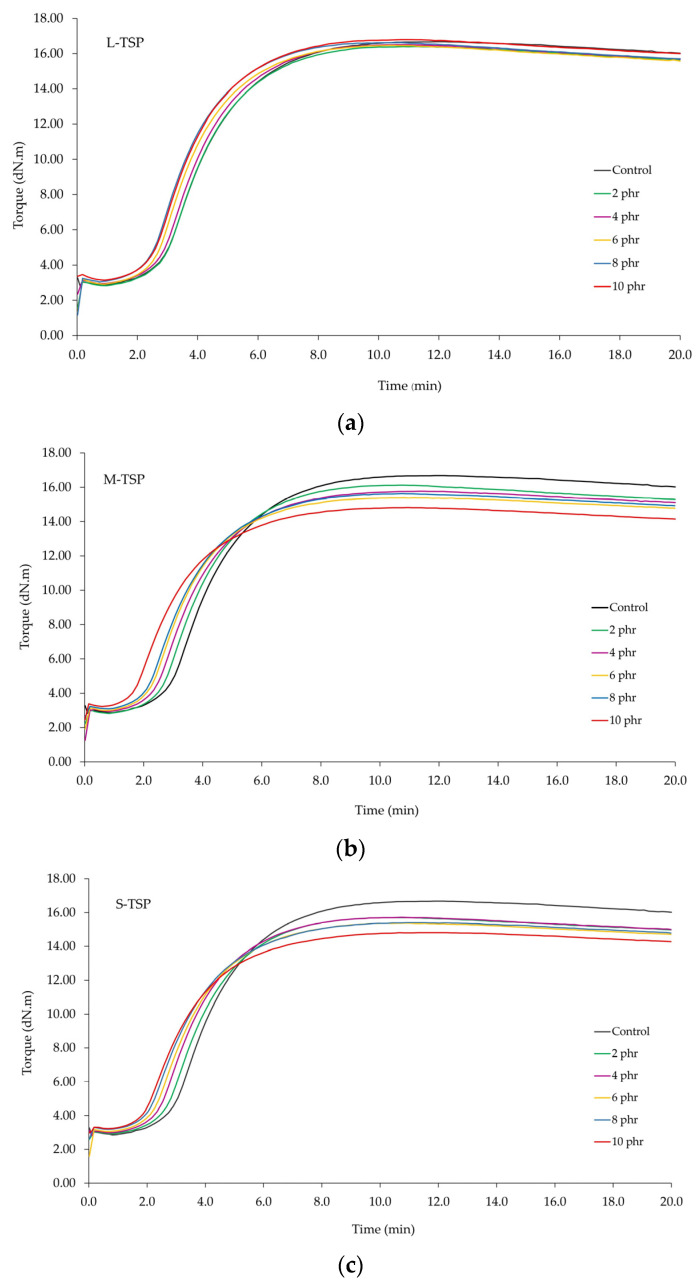
Cure curves of the rubber compounds incorporated with different TSP loadings: (**a**) L-TSP, (**b**) M-TSP, and (**c**) S-TSP.

The cure curves of the rubber compounds filled with different TSP contents are given in Figure 4. The values of scorch time (t_s1_), cure time (t_c_95), and torque difference (M_H_ − M_L_) are also tabulated in Table 4. Without the addition of TSP (control), the rubber compound had the longest scorch time (t_s1_ = 2.55 min) and cure time (t_c_95 = 7.83 min). Generally, the scorch time is widely used to indicate the safety of rubber processing because it is an induction period before the flow of the rubber compounds is affected. A long scorch time is preferred when formulating rubber compounds. However, excessively long scorch times could reduce productivity and increase production costs. In this study, the cure curves tended to shift slightly to the left with the increase in TSP content, indicating a capability to reduce the onset of the sulfur vulcanization reaction of TSP. Theoretically, the acidic substances generally retard sulfur vulcanization reactions, leading to prolonged scorch and cure times. However, the addition of TSP reduced both the scorch and cure times of the rubber compounds, despite its acidic nature. This is attributed to (1) the release of moisture from TSP, which could promote disassociation of the sulfenamide accelerator (TBBS) [38] and (2) the presence of natural proteins in TSP, which have been reported to be in a range of 1.4–3.3% [2,39].

Many published works have reported that proteins could reduce the onset of sulfur vulcanization reactions [27,40,41,42,43,44]. Alternatively, it could be said that proteins and moisture have a dominant effect on scorch and cure times over the retarding effect of acidity. However, it has been reported that, despite the reduction in cure time, the incorporation of proteins generally results in a reduction in torque difference and makes the rubber compound prone to cure reversion [27]. This explains why the torque difference, an indirect indication of the state of cure, decreased continuously with increasing TSP content [33]. In addition to proteins, the acidity of TSP may contribute to the reduction in the cure state. This phenomenon is more obvious in the M-TSP and S-TSP systems, possibly due to their higher specific surface areas and, thus, their greater reactivity to chemical reactions.

Considering the effect of particle size, it could be observed that L-TSP, which had the highest average particle size, had the least effect on the cure characteristics of the rubber compounds, whereas both M-STP and S-TSP showed stronger effects on cure characteristics, probably due to their higher specific surface area and, thus, their reactivity towards chemical reactions, as mentioned above.

#### 3.2.2. Mechanical and Aging Resistance Properties

**Figure 5:**
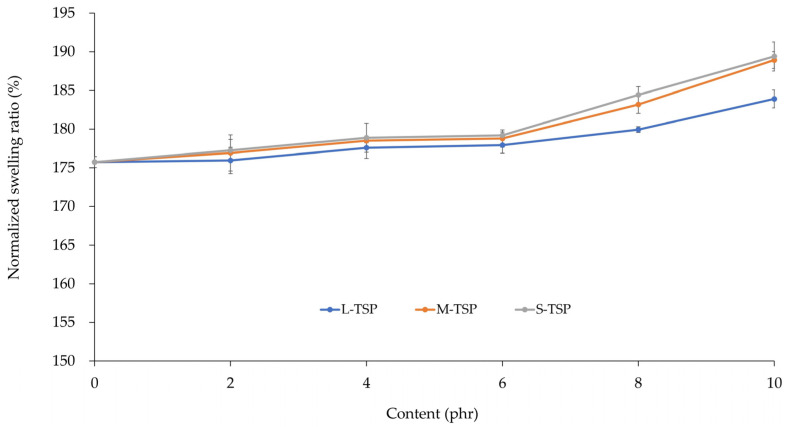
Swelling ratios of the rubber vulcanizates.

Table 5 displays the mechanical properties of the rubber vulcanizates filled with different TSP contents. The hardness of the control (without TSP) was approximately 62 Shore A. For all three TSP samples, the addition of TSP from 2 to 10 phr did not significantly affect the hardness of the vulcanizates because the TSP samples used in this study had a very large particle size. A small increase in hardness (~1–2 Shore A) was observed when a sufficient amount of TSP (≥6 phr) was added. Such an increase may be attributed to the dilution effect because the addition of solid filler will dilute the soft, deformable rubber portion, leading to increased stiffness of the rubber vulcanizates. Even though M-TSP and S-TSP had a smaller particle size than L-TSP, they did not provide a stiffer or harder vulcanizate, probably due to the reduced crosslink density as evidenced by the reduction in torque difference shown in Table 4, which was more pronounced in the M-TSP and S-TSP systems. The observations were further confirmed by the swelling test results shown in Figure 5. In theory, the swelling ratio is inversely proportional to the crosslink density of rubber. In this study, the swelling ratio of the rubber vulcanizates, after normalizing the effect of filler loading differences, tended to increase gradually with the increase in TSP loading, indicating a reduction in crosslink density in the presence of TSP. As modulus is closely related to hardness, a similar trend of the results was observed as shown in Table 5, i.e., the modulus or stress at 100% elongation (S_100_) increased slightly with the increase in TSP content. A similar explanation applies.

The tensile strength of the vulcanizate without TSP was very high (ca. 28.5 MPa). As expected, the tensile strength decreased continuously with the increase in TSP content. The reduction is more obvious in the L-TSP system due to its larger particle size and, thus, it provides the least reinforcement. It has previously been reported that large particle fillers (on the microscale) embedded in rubber matrix could act as defects, causing stress concentration and leading to premature failure or strength reduction. This explains why the reduction in strength was less obvious in the S-TSP system. Similar to tensile strength, elongation at break of the rubber vulcanizates decreased continuously with increasing TSP content, regardless of TSP grade. Such a reduction was less pronounced when the particle size of TSP was reduced. The same explanation applies.

The abrasion resistance of the rubber vulcanizates was also determined. The results are represented in terms of abrasive volume loss, as shown in Table 5. The greater the abrasive volume loss, the poorer the abrasion resistance of the rubber vulcanizates. Regardless of the TSP grade, the volume loss tended to increase with the increase in TSP content, indicating poorer abrasion resistance. This phenomenon is not uncommon because the literature has revealed a considerable reduction in abrasion resistance when inert or non-reinforcing fillers are added [45]. Deterioration of abrasion resistance in the presence of TSP may arise from the large particle size in association with poor rubber–filler interaction.

An aging resistance test was also performed. The changes in hardness, tensile strength, and elongation at break after the aging test are listed in Table 6. After being aged at 70 °C for 72 h, the hardness of the rubber vulcanizates increased approximately 3–4 Shore A due to the post-curing effect. This phenomenon is quite common in highly unsaturated rubbers, particularly natural rubber (NR), styrene butadiene rubber (SBR), and acrylonitrile butadiene rubber (NBR). The changes in tensile strength and elongation at break of all vulcanizates were also relatively low (less than 15%), indicating good aging resistance. It can be observed that the changes in mechanical properties after the aging test of the TSP-filled vulcanizates are not significantly different when compared with those of the control. The results reveal that the addition of TSP does not have a significant negative impact on the aging resistance of the rubber vulcanizates.

**Figure 6:**
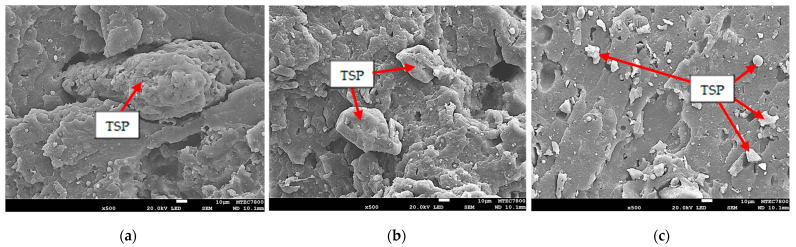
SEM images of the rubber vulcanizates incorporated with 10 phr of (**a**) L-TSP, (**b**) M-TSP, and (**c**) S-TSP.

Figure 6 shows the SEM micrographs of the rubber vulcanizates incorporated with 10 phr of TSP. Clearly, large particles of TSP were easily observed in the images. Poor interfacial interaction between TSP and NR could be expected due to the existence of interfacial voids (see Figure 6a) or pores that resulted from the TSP pulling out. The results are not uncommon because TSP is highly polar as it is composed of a lot of oxygen atoms from celluloses, proteins, and tannins, whereas NR is a non-polar hydrocarbon. The SEM results again confirm the embedment of large TSP particles in the rubber matrix and the poor rubber–TSP interaction, leading to the deterioration of mechanical properties, as previously mentioned.

**Figure 7:**
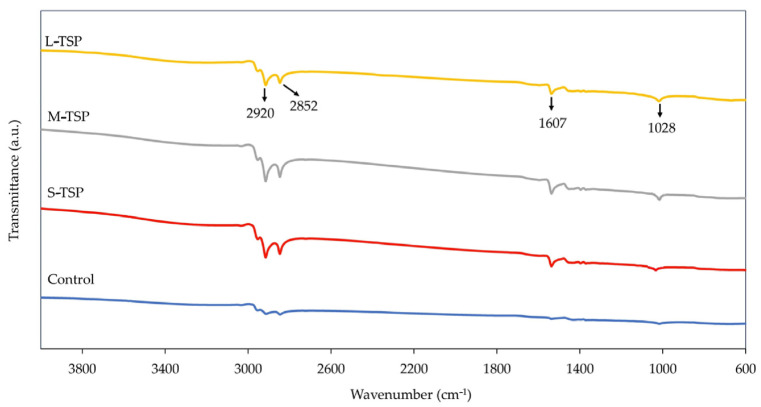
FTIR spectra of the rubber vulcanizates filled with 0 and 10 phr of TSP.

Figure 7 shows examples of FTIR spectra of the rubber vulcanizates filled with 0 and 10 phr of various TSP samples. Due to the presence of carbon black, which can absorb a wide range of spectra, including visible and infrared light, the FTIR peaks of all vulcanizates are not very clear. However, it can be seen that, compared with the control, the vulcanizates filled with TSP show additional peaks at 1607 and 1028 cm^−1^, representing the existence of protein and cellulose in the vulcanizates, respectively.

**Figure 8:**
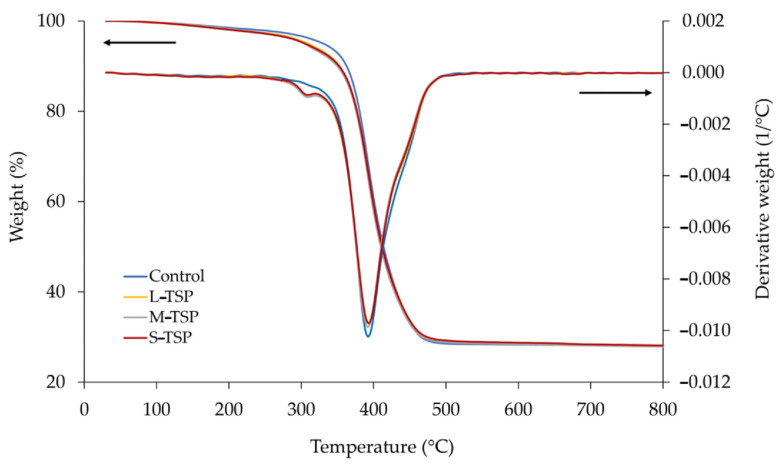
TGA and DTG curves of the rubber vulcanizates filled with 0 and 10 phr of TSP.

The TGA and DTG curves of the rubber vulcanizates filled with 0 and 10 phr of TSP are given in Figure 8. Obviously, all vulcanizates showed the maximum decomposition temperature (T_d_) at about 393 °C, which is the conventional T_d_ value of natural rubber. It could also be observed that, in the presence of TSP, the onset decomposition temperature, the temperature at which a material starts to decompose, was slightly lower. Compared with the control, the vulcanizates containing TSP showed a greater rate of weight loss at a temperature range of 300–350 °C, resulting in a small DTG peak at approximately 310 °C. The greater weight loss at this stage can be explained by the decomposition of some TSP’s components, which are less thermally stable than natural rubber. The results also revealed that the particle size of TSP had no significant effect on the thermal stability of the rubber vulcanizates. It could also be observed that all samples had a relatively high residue content (approximately 28%wt.). The residue is mainly composed of carbon black and zinc oxide, which are the main ingredients in the rubber compound. Of course, a very small amount of residue might also come from the ash obtained from the decomposition of TSP (if added).

## 4. Conclusions

The golden, fine powder of tamarind shell was prepared and characterized. It was a weak acid (pH ~ 4.5) and was composed of tannins, proteins, and celluloses. When sieved through various screens, three different grades of TSP were obtained, i.e., L-TSP, M-TSP, and S-TSP. Due to the presence of proteins and moisture, the addition of TSP caused a reduction in the onset of the vulcanization reaction, leading to a shorter scorch time and cure time, in conjunction with a decreased torque difference. These observations were more pronounced with the decrease in TSP particle size, owing to the increase in specific surface area and, thus, chemical reactivity. Although the small addition of TSP (≤10 phr) did not significantly affect the hardness of the rubber vulcanizates, it showed a negative effect on various mechanical properties, particularly tensile strength, elongation at break, and abrasion resistance. The deterioration of mechanical properties depended greatly on the TSP’s particle size, i.e., the higher the average particle size, the greater the negative effect on the mechanical properties. The results are not beyond expectation because large particle-size fillers generally act as defects for rubber, promoting premature failure. Poor interfacial interaction between hydrophilic TSP and hydrophobic NR might be another reason for such deterioration. Taken as a whole, it can be said that TSP could be used as an eco-friendly, inert biofiller in rubbers for cost-reduction and environment-protection purposes. However, it should be used with great care to avoid excessive impairment of mechanical properties. Particle size reduction and surface treatment to enhance rubber–TSP interaction are also recommended.

## Figures and Tables

**Table 1:** Suppliers of the compounding ingredients.

Ingredient	Function	Supplier
Carbon black (N330)	Reinforcing filler	Thai Carbon Black PCL. (Angthong, Thailand)
Zinc oxide (ZnO, white seal)	Inorganic activator	Thai-Lysaght Co., Ltd. (Ayutthaya, Thailand)
Stearic acid	Organic activator	Kij Paiboon Chemical LP. (Bangkok, Thailand)
N-(1,3-dimethylbutyl)-N′-phenyl-p-phenylenediamine (6PPD)	Antidegradant	Reliance Co., Ltd. (Bangkok, Thailand)
Aromatic oil	Plasticizer	Union Link Co., Ltd. (Samutprakan, Thailand)
N-tert-butylbenzothiazole-2-sulfenamide (TBBS)	Accelerator	Monflex Pte. Ltd. (Singapore)
Sulfur	Vulcanizing agent	Siam Chemical Industry Co., Ltd. (Samutprakan, Thailand)

**Table 2:** The rubber formulas.

Ingredient	Content (Parts per Hundred Rubber: phr)
Natural rubber (STR 5 L)	100
Zinc oxide	3
Stearic acid	1
TSP *	0, 2, 4, 6, 8, and 10
Carbon black (N330)	40
6PPD	1
Aromatic oil	2
TBBS	1
Sulfur	2

* S-TSP, M-TSP, and L-TSP.

**Table 3:** Average particle size, moisture content, density, and pH of the TSP samples.

Sample	Average Particle Size (µm)	Moisture Content (%)	Density (g/cm^3^)	pH
L-TSP	191.5 ± 0.1	5.7 ± 0.3	1.56 ± 0.04	4.5 ± 0.1
M-TSP	29.8 ± 0.1	6.7 ± 0.4		
S-TSP	17.2 ± 0.0	6.7 ± 0.3		

**Table 4:** Cure characteristics of the rubber compounds.

Compound	TSP Content (phr)	Scorch Time, t_s1_, (min)	Optimum Cure Time, t_c_95, (min)	Torque Difference, M_H_ − M_L_, (dN.m)
NR/Control	0	2.6	7.8	13.9
NR/L-TSP	2	2.5	7.6	13.6
	4	2.5	7.4	13.6
	6	2.4	7.2	13.6
	8	2.2	7.0	13.6
	10	2.3	7.2	13.7
NR/M-TSP	2	2.4	7.3	13.3
	4	2.2	7.3	12.9
	6	2.1	7.0	12.4
	8	1.9	7.1	12.7
	10	1.7	6.0	11.6
NR/S-TSP	2	2.4	7.2	12.8
	4	2.1	7.2	12.9
	6	2.1	7.2	12.3
	8	2.0	7.3	12.2
	10	1.9	7.2	11.7

**Table 5:** Mechanical properties of the rubber vulcanizates.

Compound	Content (phr)	Hardness (Shore A)	Tensile Strength (MPa)	Stress at 100 Elongation (MPa)	Elongation at Break (%)	Volume Loss (mm^3^)
NR/Control	0	61.6 ± 0.2	28.5 ± 0.9	3.13 ± 0.13	487 ± 11	124.7 ± 0.5
NR/L-TSP	2	61.9 ± 0.2	25.9 ± 0.4	3.17 ± 0.14	430 ± 14	119.8 ± 2.2
	4	62.1 ± 0.4	24.0 ± 0.3	3.22 ± 0.19	409 ± 12	125.2 ± 1.8
	6	63.2 ± 0.3	20.1 ± 0.4	3.27 ± 0.15	393 ± 13	129.8 ± 0.8
	8	62.5 ± 0.3	20.9 ± 0.2	3.27 ± 0.16	374 ± 15	131.7 ± 0.7
	10	63.5 ± 0.2	19.1 ± 0.1	3.30 ± 0.22	372 ± 8	135.4 ± 0.7
NR/M-TSP	2	62.4 ± 0.5	26.3 ± 0.4	3.23 ± 0.20	437 ± 11	126.4 ± 3.4
	4	62.4 ± 0.4	26.3 ± 0.7	3.19 ± 0.07	429 ± 11	129.5 ± 2.6
	6	62.9 ± 0.2	25.2 ± 0.4	3.24 ± 0.13	434 ± 7	133.7 ± 1.0
	8	63.0 ± 0.3	24.1 ± 0.2	3.25 ± 0.24	427 ± 16	138.4 ± 2.1
	10	62.8 ± 0.2	22.8 ± 0.3	3.29 ± 0.19	417 ± 16	145.7 ± 2.8
NR/S-TSP	2	62.4 ± 0.3	27.8 ± 1.2	3.22 ± 0.04	447 ± 15	128.3 ± 1.6
	4	62.5 ± 0.2	27.3 ± 0.1	3.23 ± 0.09	461 ± 10	128.5 ± 4.4
	6	62.8 ± 0.2	25.8 ± 0.7	3.26 ± 0.11	438 ± 20	134.6 ± 2.1
	8	63.1 ± 0.2	24.7 ± 0.2	3.28 ± 0.08	426 ± 16	140.7 ± 2.6
	10	62.3 ± 0.2	23.9 ± 0.3	3.38 ± 0.25	397 ± 6	145.5 ± 4.0

**Table 6:** Properties of the rubber vulcanizates after the aging test.

Compound	Content (phr)	Property after the Aging Test			Property Change		
		Hardness (Shore A)	Tensile Strength (MPa)	Elongation at Break (%)	Hardness (Shore A)	Tensile Strength (%)	Elongation at Break (%)
NR/Control	0	64.9 ± 0.4	27.1 ± 0.4	448 ± 14	3.3	−4.9	−8.0
NR/L-TSP	2	65.6 ± 0.2	23.5 ± 0.6	408 ± 4	3.7	−9.3	−5.1
	4	66.1 ± 0.2	22.8 ± 0.4	395 ± 14	4.0	−5.0	−3.4
	6	66.2 ± 0.4	20.2 ± 0.4	356 ± 14	3.0	0.5	−9.4
	8	66.5 ± 0.4	20.2 ± 0.6	354 ± 13	4.0	−3.3	−5.3
	10	66.9 ± 0.2	18.5 ± 0.4	328 ± 8	3.4	−3.1	−11.8
NR/M-TSP	2	65.2 ± 0.3	26.8 ± 0.8	433 ± 11	2.8	1.9	−0.9
	4	65.4 ± 0.2	25.9 ± 0.6	414 ± 15	3.0	−1.5	−3.5
	6	65.7 ± 0.3	24.8 ± 0.2	410 ± 9	2.8	−1.6	−5.5
	8	66.5 ± 0.3	23.8 ± 0.2	409 ± 9	3.5	−1.2	−4.2
	10	66.0 ± 0.3	23.2 ± 0.2	409 ± 15	3.2	1.8	−1.9
NR/S-TSP	2	65.8 ± 0.4	27.6 ± 0.5	438 ± 15	3.4	−0.7	−2.0
	4	65.9 ± 0.3	26.5 ± 0.4	404 ± 15	3.4	−2.9	−12.4
	6	65.9 ± 0.3	25.9 ± 0.5	400 ± 20	3.1	0.4	−8.7
	8	66.2 ± 0.2	24.2 ± 0.6	372 ± 3	3.1	−2.0	−12.7
	10	66.3 ± 0.4	23.9 ± 0.2	349 ± 6	4.0	0.0	−12.1

## Data Availability

The data presented in this study are available on request from the corresponding author.
